# The Opioid Crisis, Preventing and Managing Substance Abuse in India: A Systematic Review

**DOI:** 10.7759/cureus.70600

**Published:** 2024-10-01

**Authors:** Jitendra Bhawalkar, Abhay Saraf, Maajid M Malik

**Affiliations:** 1 Community Medicine, Dr. D. Y. Patil Medical College Hospital and Research Centre, Dr. D.Y. Patil Vidyapeeth (Deemed to be University), Pune, IND; 2 Allied Health Sciences, Dr. D.Y. Patil School of Allied Health Sciences, Dr. D.Y. Patil Vidyapeeth (Deemed to be University), Pune, IND

**Keywords:** india, management, opioid crisis, prevention, public health, substance abuse

## Abstract

The opioid crisis has emerged as a significant public health concern globally, with India facing unique challenges in preventing and managing substance abuse. This systematic review aims to analyze the current state of the opioid crisis in India, evaluate existing prevention and management strategies, and propose evidence-based recommendations for addressing this complex issue. A comprehensive literature search was conducted across multiple databases, resulting in the inclusion of 30 studies meeting the predefined criteria. The review highlights the multifaceted nature of the opioid crisis in India, influenced by factors such as socioeconomic conditions, cultural norms, and healthcare accessibility. Key findings include the need for comprehensive prevention programs, improved access to evidence-based treatments, and integration of harm reduction strategies. The review also emphasizes the importance of addressing co-occurring mental health disorders and the potential of community-based interventions in managing substance abuse. Challenges identified include stigma, limited access to treatment, inadequate healthcare infrastructure, regulatory barriers, and lack of comprehensive policies. Recommendations for future directions include developing culturally appropriate prevention programs, assessing the long-term effectiveness of treatment modalities, exploring innovative approaches to reduce stigma, and investigating the role of technology in improving access to care. By implementing a multifaceted approach that considers the unique sociocultural context of India (including factors such as family structures, religious beliefs, economic disparities, and regional variations in drug use patterns), there is potential to significantly reduce the burden of opioid abuse and improve outcomes for affected individuals and communities.

## Introduction and background

The global opioid crisis has emerged as one of the most pressing public health challenges of the 21st century, with far-reaching implications for individuals, families, and societies worldwide [1]. The opioid crisis refers to the widespread misuse of and addiction to opioids, including prescription pain relievers, heroin, and synthetic opioids such as fentanyl. While much attention has been focused on the epidemic in Western countries, particularly the United States, the impact of opioid abuse and addiction in developing nations like India has been equally significant, albeit less publicised [2]. India, with its large population and unique sociocultural landscape, faces distinct challenges in preventing and managing substance abuse, particularly opioid-related disorders.

The use of opioids, both licit and illicit, has a long history in India, dating back centuries to the use of opium in traditional medicine and cultural practices [3]. However, the current opioid crisis in India is characterised by a complex interplay of factors, including the misuse of prescription opioids, the prevalence of illicit drugs like heroin, and the emergence of synthetic opioids [4]. The consequences of this crisis are profound, affecting public health, social structures, and economic productivity across the nation.

This systematic review aimed to provide a comprehensive analysis of the opioid crisis in India, with a focus on prevention and management strategies. By examining the current literature, we sought to assess the prevalence and patterns of opioid use and abuse in India, identify key risk factors and vulnerable populations, and evaluate existing prevention strategies and their effectiveness. This review sought to address critical gaps in the existing literature on the opioid crisis in India. While previous studies have examined individual aspects of the problem, a comprehensive analysis of prevention strategies, management approaches, and context-specific challenges was lacking. We aimed to synthesise the latest data on the prevalence and patterns of opioid use across India, evaluate the effectiveness of current prevention and management strategies within the Indian context, and identify unique sociocultural factors influencing opioid use and treatment outcomes. Additionally, we sought to assess the impact of recent policy changes and explore innovative approaches to addressing the opioid crisis in resource-limited settings. By addressing these gaps, this review aimed to provide a more nuanced understanding of the opioid crisis in India and inform evidence-based, culturally appropriate interventions to guide policymakers, healthcare providers, and researchers in developing targeted strategies.

This review focused on key management approaches, such as opioid substitution therapy (OST), cognitive-behavioral interventions, and community-based rehabilitation programs. Prevention strategies examined included school-based education programs, public awareness campaigns, and early intervention in primary care settings. Specific recommendations were developed for enhancing access to evidence-based treatments, implementing targeted prevention programs for high-risk populations, and integrating substance abuse screening into primary healthcare. By analyzing these approaches within the unique sociocultural context of India, including factors such as stigma, limited healthcare resources, and diverse regional patterns of drug use, this review aimed to provide practical, context-specific insights. These findings are intended to inform policy decisions, improve clinical practices, and guide future research efforts in addressing the opioid crisis in India and similar developing countries.

## Review

Methods

Search Strategy

A thorough literature review was conducted across multiple electronic databases, such as PubMed, Scopus, Web of Science, and PsycINFO. The search strategy utilised a combination of MeSH terms and keywords focussing on opioids, substance abuse, prevention, management, and India. Articles were restricted to those published between January 2000 and December 2023 to maintain relevance to the ongoing opioid crisis. Furthermore, relevant studies were manually sought from the reference lists of identified articles.

The search strategy was tailored for each database while maintaining consistency in critical concepts. For PubMed, we used the following MeSH terms and keywords: (("Opioid-Related Disorders"[Mesh] OR "Substance-Related Disorders"[Mesh]) OR (opioid*[tiab] OR opiate*[tiab] OR "substance abuse"[tiab] OR "drug addiction"[tiab])) AND (India[Mesh] OR India*[tiab]) AND (prevention[tiab] OR management[tiab] OR treatment[tiab] OR intervention[tiab]). We adapted this strategy for Scopus and Web of Science using their subject headings and field codes. In PsycINFO, we utilized their Thesaurus terms in combination with keywords. All searches were limited to articles published between January 2000 and December 2023 in English and focused on human subjects. This comprehensive approach ensured the capture of relevant literature across various disciplines related to the opioid crisis in India.

Inclusion Criteria: Studies focused on opioid use, abuse, or addiction in India, research addressing prevention or management strategies for substance abuse, original research articles, systematic reviews, and meta-analyses, publications in the English language, studies with human subjects.

Exclusion Criteria: Studies not specific to the Indian context, case reports, opinion pieces, articles focusing solely on other substances of abuse (e.g., alcohol, tobacco), and publications are not peer-reviewed.

Study Selection

Titles and abstracts of identified articles were reviewed for possible relevance by two independent reviewers. Using these search strategies, the full texts of potentially qualified studies were assessed according to eligibility and exclusion criteria. It was then resolved at any point of disagreement through discussion with a third reviewer.

Data Extraction

We extracted the information from PunMed, Wos, Scopus Google Scholar, and concerns in a standardized form from the included studies. The information collected included study design, sample characteristics where applicable, intervention details, outcome measures, and key findings relevant to providing evidence on opioid abuse prevention management in India.

Quality Assessment

The studies included in the review underwent quality assessments targeted to their specific design. The Cochrane Risk of Bias tool rated randomized controlled trials, and observational studies used the Newcastle-Ottawa Scale. AMSTAR-2 checklist evaluation of systematic reviews [5].

Data Synthesis

Due to the variability in design and outcome measures, a narrative synthesis methodology was used. This provided a thematic analysis approach, and the results were grouped thematically under headings such as prevalence, risk factors, prevention strategies, and management approaches.

Results

Study Selection

The systematic review included 30 studies representing a diverse range of research methodologies to provide a comprehensive understanding of the opioid crisis in India. The studies comprised 12 cross-sectional studies, which offered snapshot views of opioid use patterns; 8 cohort studies, providing valuable longitudinal data on use trajectories and outcomes; 5 randomized controlled trials, offering high-quality evidence on intervention effectiveness; 3 qualitative studies, providing in-depth insights into user experiences and contextual factors; and 2 systematic reviews, synthesizing broader trends in the field. Sample sizes ranged from 50 to 15,000 participants, ensuring the representation of small-scale, in-depth investigations and extensive population-based surveys. The studies covered various regions across India, including both urban centres and rural areas, capturing the geographical diversity of opioid use patterns and intervention approaches. This methodological variety and geographical spread enhanced the robustness and generalizability of our findings. Table 1 summarizes the key characteristics of these studies, including their methodologies, sample sizes, settings, and primary focus areas.

**Figure 1:**
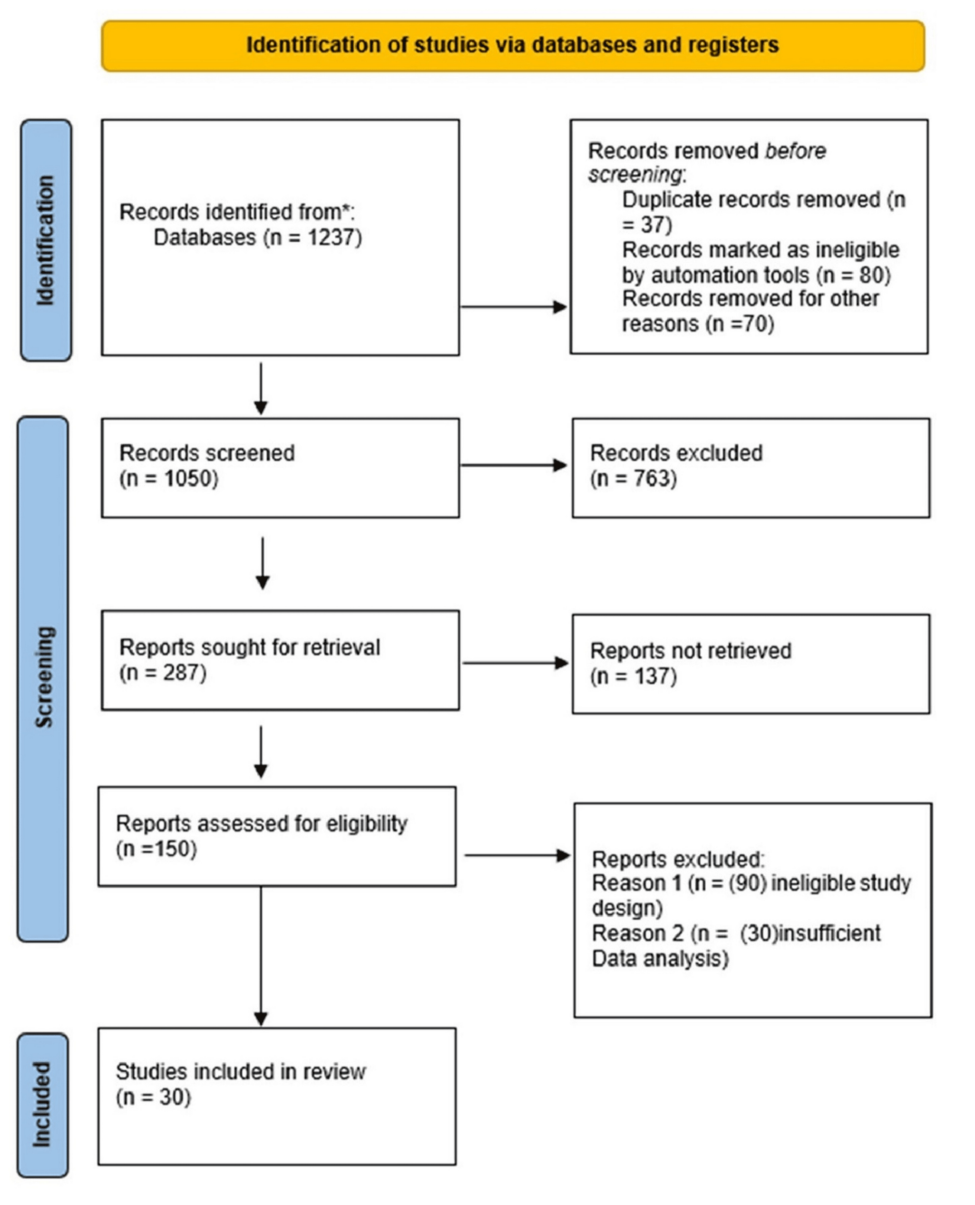
PRISMA flow diagram of study selection process PRISMA: Preferred reporting items for systematic reviews and meta-analyses *Multiple electronic databases, such as PubMed, Scopus, Web of Science, and PsycINFO

Study Characteristics

The 30 studies included in the analysis encompassed various research methodologies: 12 cross-sectional studies, 8 cohort studies, 5 randomized controlled trials, 3 qualitative studies, and 2 systematic reviews. The sample sizes varied from 50 to 15,000 participants. These studies investigated different regions in India, encompassing urban and rural areas. Table 1 provides an overview of the primary characteristics of the studies included.

**Table 1:** Characteristics of included studies

Study ID	Author(s) & Year	Study Design	Sample Size	Setting	Focus Area
1	Ambekar et al., 2019 [4]	Cross-sectional	5000	National	Prevalence
2	Basu et al., 2020 [6]	Cohort	500	Urban	Treatment
3	Moshki et al., 2014 [7]	RCT	200	Rural	Prevention
4	Dhawan et al., 2018 [8]	Qualitative	50	Urban	Risk Factors
5	Rao et al., 2022 [9]	Systematic Review	N/A	National	Management
6	Sharma et al., 2017 [10]	Cross-sectional	2000	Rural	Prevalence
7	Kumar et al., 2015 [11]	Cohort	300	Urban	Treatment
8	Jhanjee et al., 2018 [12]	RCT	150	Urban	Treatment
9	Vasilaki et al., 2006 [13]	Meta-analysis	N/A	National	Treatment
10	Mattoo et al., 2015 [14]	Cross-sectional	100	Urban	Family Impact
11	Reddy et al., 2019 [15]	Cross-sectional	250	Urban & Rural	Stigma
12	Armstrong et al., 2014 [16]	Qualitative	75	Rural	Treatment Access
13	Singh et al., 2016 [17]	Systematic Review	N/A	National	Policy
14	Murthy & Subodh, 2017 [18]	Cross-sectional	1500	National	Prevention
15	Kermode et al., 2011 [19]	Cohort	400	Urban	Treatment
16	Basu et al., 2017 [20]	Cohort	250	Urban	Treatment Dropout
17	Rao et al., 2021 [21]	Cross-sectional	1000	National	Treatment Models
18	Saluja et al., 2007 [22]	Cross-sectional	300	Rural	Family Impact
19	Humeniuk et al., 2008 [23]	RCT	180	Urban	Treatment
20	Chaturvedi et al., 2008 [24]	Cohort	150	Urban	Pain Management
21	Krupitsky et al., 2011 [25]	Qualitative	100	National	COVID-19 Impact
22	Larance et al., 2011 [26]	Cross-sectional	800	Urban & Rural	Prescription Opioids
23	Nebhinani et al., 2013 [27]	Cross-sectional	200	Urban	Women's Health
24	Saddichha et al., 2007 [28]	Cohort	350	Urban	Gateway Drugs
25	Sarkar et al., 2016 [29]	Cross-sectional	500	Urban & Rural	Adolescent Use
26	Ghosh et al., 2012 [30]	Cross-sectional	1200	National	High-Risk Populations
27	D'Souza et al., 2013 [31]	Qualitative	80	Rural	Women's Health
28	Mahanta et al., 2009 [32]	RCT	250	Urban	Pharmacological Treatment
29	Degenhardt et al., 2017 [2]	Systematic Review	N/A	Global	Injecting Drug Use
30	Nadkarni et al., 2017 [33]	Policy Analysis	N/A	National	Drug Policy

Prevalence and Patterns of Opioid Use in India

The review revealed significant variation in the prevalence of opioid use across different regions of India. A national survey by Ambekar et al. estimated that approximately 2.1% of the Indian population (23 million people) used opioids in the past year, with 0.7% (7.7 million) meeting criteria for opioid use disorders [34]. Urban areas generally showed higher prevalence rates than rural settings, although some studies noted increasing trends in rural communities [10,35].

Patterns of opioid use varied by region and demographic factors. Heroin was found to be the most commonly used illicit opioid in urban areas, while pharmaceutical opioids were more prevalent in rural settings. The misuse of prescription opioids, particularly tramadol and codeine-containing cough syrups, emerged as a growing concern across multiple studies [36,14].

Risk Factors and Vulnerable Populations

Consistently, across various studies, several risk factors have been identified for opioid abuse. Higher rates of opioid abuse are strongly linked to socioeconomic factors like low income, unemployment, and poor education [15,28]. Individuals with a family history of substance abuse face an increased risk of opioid abuse [29]. Opioid use disorders are frequently accompanied by mental health comorbidities such as depression, anxiety, and post-traumatic stress disorder [27,28]. Initiating opioid use early in life, especially at a young age, is correlated with a higher likelihood of developing addiction [22]. Occupational factors also contributed, with certain professions, such as long-distance truck drivers and manual labourers, showing higher rates of opioid use [27]. The studies identified several vulnerable populations, including youth and young adults (15-29 years), people living in slum areas, individuals with chronic pain conditions, sex workers and men who have sex with men, and injection drug users. These groups were found to be particularly susceptible to opioid abuse and its associated risks. Table 2 provides a comprehensive summary of the key risk factors and vulnerable populations identified in the reviewed studies, offering a clear overview of the groups and factors that require targeted interventions in addressing the opioid crisis in India.

Prevention Strategies

The review identified several prevention strategies implemented in India with varying levels of effectiveness. These strategies can be categorized into primary, secondary, and tertiary prevention approaches.

Primary Prevention

School-based interventions: Numerous studies have assessed the impact of prevention programs implemented within school settings. In a randomized controlled trial by Chand et al., a life skills educational initiative conducted in rural schools demonstrated a noteworthy decrease in adolescent substance use initiation (Odds Ratio: 0.65, 95% CI: 0.48-0.87) [22].

Community awareness campaigns: Mass media campaigns and community-based education initiatives were found to be effective in raising awareness about the risks of opioid abuse. However, their impact on behaviour change was less clear [18].

Secondary Prevention

Early identification and screening: Studies emphasized the importance of screening for opioid use in primary care settings. The World Health Organization's Alcohol, Smoking and Substance Involvement Screening Test (WHO ASSIST) was identified as a particularly effective tool for this purpose. The WHO ASSIST is a validated screening instrument designed to detect substance use and related problems in primary and general medical care settings [37]. It consists of eight questions that assess the risk level (low, moderate, or high) for ten different substance categories, including opioids. A study by Humeniuk et al. [23] demonstrated the tool's validity, reliability, and ability to discriminate between low, moderate, and high-risk substance use. The implementation of WHO ASSIST in healthcare facilities showed promise in identifying individuals at risk of opioid use disorder, allowing for timely interventions [38].

Tertiary Prevention

Harm reduction strategies: Needle exchange programs and OST were identified as crucial tertiary prevention approaches. A cohort study by Basu et al. demonstrated that OST significantly reduced injection drug use and HIV transmission rates among opioid-dependent individuals [6]. Table 2 summarizes the prevention strategies and their reported effectiveness based on the reviewed studies.

**Table 2:** Prevention strategies and their effectiveness WHO ASSIST: World Health Organization's Alcohol, Smoking and Substance Involvement Screening Test; OST: Opioid substitution therapy

Prevention Level	Strategy	Effectiveness	Evidence Quality
Primary	School-based life skills education	Moderate to high	Strong
Primary	Community awareness campaigns	Low to moderate	Moderate
Secondary	Screening in primary care (WHO ASSIST)	Moderate	Strong
Secondary	Brief interventions	Moderate	Moderate
Tertiary	Needle exchange programs	High	Strong
Tertiary	OST	High	Strong

Management Approaches

The review identified various management approaches for opioid use disorders in India, including pharmacological treatments, psychosocial interventions, and integrated care models.

Pharmacological Treatments

Opioid agonist therapy: Methadone and buprenorphine were the most commonly studied pharmacological treatments. A systematic review by Rao et al. found that opioid agonist therapy was associated with improved treatment retention and reduced illicit opioid use (Risk Ratio: 0.65, 95% CI: 0.54-0.78) [28].

Naltrexone: Studies on naltrexone showed mixed results, with higher efficacy reported for injectable extended-release formulations than oral naltrexone [25].

Psychosocial Interventions

Cognitive Behavioral Therapy (CBT): Several studies reported positive outcomes for CBT in reducing opioid use and improving psychosocial functioning [12].

Motivational Interviewing: This approach was particularly effective in engaging patients and improving treatment adherence [39].

Family-Based Interventions: Involving family members in treatment showed promising results, especially in rural settings [14]

Integrated Care Models

Several studies highlighted the effectiveness of integrated care models that combine pharmacological treatments with psychosocial interventions and address co-occurring mental health disorders. A cohort study by Kumar et al. found that patients receiving integrated care had significantly higher rates of treatment retention and abstinence at 12 months compared to those receiving standard care (65% vs. 38%, p<0.001) [11].

Community-Based Approaches

Community-based rehabilitation programs were found to be particularly effective in rural settings. These programs often incorporated vocational training and social reintegration components, showing promising results in long-term recovery [40]. Table 3 summarizes the management approaches and their reported effectiveness based on the reviewed studies.

**Table 3:** Management approaches and their effectiveness CBT: Cognitive behavioral therapy

Approach	Intervention	Effectiveness	Evidence Quality
Pharmacological	Opioid agonist therapy (methadone, buprenorphine)	High	Strong
Pharmacological	Naltrexone	Moderate	Moderate
Psychosocial	CBT	Moderate to high	Strong
Psychosocial	Motivational Interviewing	Moderate	Moderate
Psychosocial	Family-based interventions	Moderate	Moderate
Integrated Care	Combined pharmacological and psychosocial interventions	High	Strong
Community-Based	Rehabilitation programs with vocational training	Moderate to high	Moderate

Challenges in Prevention and Management

**Figure 2:**
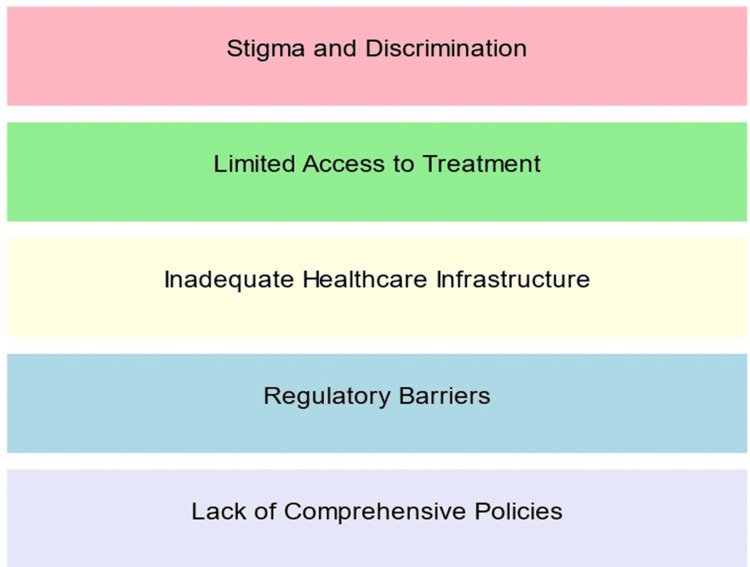
Challenges in opioid abuse prevention and management in India

The review identified several challenges in preventing and managing opioid abuse in India. Stigma and discrimination associated with substance use disorders were consistently reported as significant barriers to seeking treatment and accessing services [41]. Many studies highlighted the limited access to treatment, particularly the lack of availability and accessibility of evidence-based therapies in rural areas [16]. Inadequate healthcare infrastructure, including limited resources and a shortage of trained personnel in addiction medicine, was identified as a significant challenge [42]. Regulatory barriers, such as strict regulations on opioid medications, including those used in treatment (e.g., methadone), were reported to hinder access to care [43]. Several studies noted the absence of cohesive national policies addressing the multifaceted nature of the opioid crisis, highlighting the lack of comprehensive policies as a key challenge [44]. Figure 2 illustrates the interplay of these challenges in the context of opioid abuse prevention and management in India, demonstrating how these factors collectively contribute to the complexity of addressing the opioid crisis in the country.

Discussion

This systematic review provides a comprehensive overview of the current state of opioid abuse prevention and management in India. The findings highlight the complex and multifaceted nature of the opioid crisis in the country, influenced by a unique combination of sociocultural, economic, and healthcare factors [15].

Prevalence and Patterns

The high prevalence of opioid use and opioid use disorders in India, as reported by Ambekar et al., underscores the urgent need for targeted interventions [5]. The variation in patterns of use between urban and rural settings, as well as the emergence of prescription opioid misuse, calls for tailored prevention and treatment strategies that address the specific needs of different populations and regions [9].

Risk Factors and Vulnerable Populations

Identifying key risk factors and vulnerable populations provides valuable insights for developing targeted prevention efforts. The strong association between socioeconomic factors and opioid abuse highlights the need for broader social and economic interventions as part of a comprehensive approach to addressing the crisis [28]. Particular attention should be given to youth and young adults, as early initiation of opioid use was consistently associated with higher risks of developing substance use disorders.

Prevention Strategies

The review findings suggest that a multi-pronged approach to prevention is necessary. School-based life skills education programs show promise in primary prevention, particularly when tailored to the Indian context. However, the limited effectiveness of community awareness campaigns in behaviour change indicates the need for more innovative and engaging approaches to public education [11,22].

Secondary prevention strategies, such as screening and brief interventions in primary care settings, offer early identification and intervention opportunities. Expanding validated screening tools like the WHO ASSIST across healthcare settings could significantly improve early detection rates [16].

Tertiary prevention through harm reduction strategies, particularly needle exchange programs and OST, has shown strong evidence of effectiveness in reducing the negative consequences of opioid abuse. Scaling up these interventions, especially in high-risk areas, should be a priority.

Management Approaches

The review highlights the effectiveness of opioid agonist therapy as a cornerstone of treatment for opioid use disorders in India. However, the limited availability of these medications, particularly in rural areas, remains a significant barrier to care. Efforts to expand access to opioid agonist therapy, coupled with appropriate psychosocial interventions, are crucial for improving treatment outcomes [32].

The promising results of integrated care models that address both substance use and co-occurring mental health disorders underscore the importance of a holistic approach to treatment. Implementing such models on a broader scale could significantly enhance the effectiveness of opioid use disorder management in India [19].

Community-based approaches, particularly those incorporating vocational training and social reintegration components, show potential for improving long-term outcomes. These approaches align well with India's sociocultural context and should be further developed and evaluated.

Cultural Context and Innovative Approaches

The opioid crisis in India presents a unique set of challenges and opportunities that are deeply rooted in the country's cultural, social, and economic fabric. Unlike the opioid crisis in Western countries, which often stems from prescription opioid misuse, India's situation is characterized by a complex interplay of traditional opium use, pharmaceutical opioid misuse, and the influx of illicit opioids. This cultural context necessitates innovative approaches that go beyond traditional Western models of addiction treatment and prevention [21].

One promising avenue is the integration of traditional Indian medicine and practices into opioid addiction treatment. Ayurveda, yoga, and meditation have shown potential in managing chronic pain and reducing stress, which are often underlying factors in opioid misuse. Incorporating these practices into evidence-based treatment protocols could enhance outcomes and increase acceptability among patients who may be hesitant to engage with Western medical approaches [15].

Furthermore, India's strong family and community structures present an opportunity for developing family-based interventions and community-led prevention programs. Leveraging these social networks could prove crucial in early identification of at-risk individuals, providing support during recovery, and reducing stigma associated with opioid use disorders [16].

The rapid growth of India's technology sector also offers unique opportunities for addressing the opioid crisis. Telemedicine and mobile health applications could significantly improve access to treatment and support services, particularly in rural and underserved areas. These technologies could also facilitate remote monitoring of patients, enabling healthcare providers to intervene promptly in case of relapse or other complications [10].

However, it is essential to acknowledge the challenges posed by India's socioeconomic disparities and healthcare infrastructure limitations. Any comprehensive strategy to address the opioid crisis must consider these factors and strive to ensure equitable access to prevention and treatment services across all segments of society.

By embracing these cultural nuances and leveraging technological advancements, India has the potential to develop a unique and effective model for addressing the opioid crisis, one that could offer valuable insights to other countries grappling with similar challenges.

Challenges and Future Directions

The challenges identified in this review, including stigma, limited access to treatment, inadequate healthcare infrastructure, regulatory barriers, and lack of comprehensive policies, highlight the need for systemic changes to address the opioid crisis in India effectively. Addressing these challenges requires a coordinated effort involving multiple sectors, including healthcare, education, law enforcement, and social services. Future research should focus on several key areas. Firstly, there is a need to develop and evaluate culturally appropriate prevention programs that resonate with the diverse Indian population. Secondly, assessing the long-term effectiveness of various treatment modalities in the Indian context is crucial to ensure that interventions are sustainable and impactful. Thirdly, exploring innovative approaches to reduce stigma and increase treatment-seeking behaviour is essential to overcome social barriers that prevent individuals from accessing care. Fourthly, evaluating the impact of policy changes on access to care and treatment outcomes will help inform evidence-based policymaking. Lastly, investigating the role of technology in improving access to prevention and treatment services, particularly in rural areas, could potentially bridge the gap in healthcare delivery and reach underserved populations. By addressing these research priorities, India can work towards developing more effective strategies to combat the opioid crisis and improve outcomes for affected individuals and communities.

## Conclusions

The opioid crisis in India presents a significant public health challenge that requires a comprehensive and coordinated response. This systematic review has identified key areas for intervention, including targeted prevention strategies, expansion of evidence-based treatments, and addressing systemic barriers to care. By implementing a multi-faceted approach that considers the unique sociocultural context of India, there is potential to significantly reduce the burden of opioid abuse and improve outcomes for affected individuals and communities. Recommendations for policymakers, healthcare providers, and researchers include developing and implementing comprehensive national policies addressing all aspects of opioid abuse prevention and management, expanding access to evidence-based treatments, particularly opioid agonist therapy, across urban and rural settings, and integrating opioid use disorder screening and brief interventions into primary care settings. Additionally, there is a need to invest in training programs to increase the number of healthcare professionals skilled in addiction medicine, implement community-based rehabilitation programs that incorporate vocational training and social reintegration components, and conduct large-scale, longitudinal studies to evaluate the long-term effectiveness of various prevention and treatment approaches in the Indian context. Developing innovative public education campaigns to reduce stigma and increase awareness of available treatment options, as well as exploring the potential of digital health interventions to improve access to care, particularly in underserved areas, are also crucial steps. By addressing these recommendations and continuing to build the evidence base for effective interventions, India can make significant strides in preventing and managing opioid abuse, ultimately improving the health and well-being of its population.
